# Association between blood cadmium and prevalent coronary heart disease in NHANES 2013 to 2014: A cross-sectional study with machine-learning analyses

**DOI:** 10.1097/MD.0000000000049554

**Published:** 2026-07-03

**Authors:** Hui Guo, Yi Li, Meng Liu, Ye Zhou, Mei Hong

**Affiliations:** a Department of Cardiology, The Second Affiliated Hospital of Nanjing Medical University, Nanjing, China.

**Keywords:** blood cadmium, coronary heart disease, cross-sectional study, machine learning, NHANES, SHAP, smoking confounding

## Abstract

Cadmium is a toxic environmental metal, but its association with prevalent coronary heart disease (CHD) remains uncertain. We examined whether blood cadmium concentrations were associated with prevalent CHD in US adults and explored machine-learning model performance. This cross-sectional study used data from the National Health and Nutrition Examination Survey 2013 to 2014. Adults aged ≥20 years with complete data on self-reported CHD, blood cadmium, and covariates were included. Blood cadmium was measured by inductively coupled plasma mass spectrometry. Survey-weighted logistic regression was used to assess associations between blood cadmium and prevalent CHD. Sensitivity analyses additionally adjusted for serum cotinine and were repeated in never smokers. Ten machine-learning algorithms were developed to assess discrimination, calibration, and feature importance. Among 2219 participants, higher blood cadmium concentrations were associated with higher odds of prevalent CHD. In the fully adjusted model, each 1-unit increase in ln-transformed blood cadmium was associated with prevalent CHD (odds ratio, 1.38; 95% confidence interval, 1.05–1.81). Compared with the lowest quartile, the highest quartile was associated with higher odds of prevalent CHD (odds ratio, 1.95; 95% confidence interval, 1.25–3.04). The association remained after additional adjustment for serum cotinine but was attenuated in never smokers. The multilayer perceptron showed the best overall discrimination and calibration; age ranked first and blood cadmium ranked second by mean absolute Shapley Additive Explanations value. Higher blood cadmium concentrations were associated with higher odds of prevalent CHD in this cross-sectional analysis. These findings should be interpreted cautiously because residual smoking-related confounding remains possible and the CHD outcome was self-reported.

## 1. Introduction

Coronary heart disease (CHD) remains a major contributor to global morbidity, mortality, and health care burden.^[1]^ Although established cardiometabolic and behavioral factors remain central to CHD prevention, environmental exposures are increasingly recognized as relevant contributors to cardiovascular risk.^[2,3]^ Among these, toxic metal contaminants have attracted growing attention because they are widespread, potentially modifiable, and biologically plausible contributors to vascular injury and atherosclerotic disease.^[2,3]^

Cadmium is a toxic metal to which humans are exposed through tobacco smoke, industrial emissions, and contaminated food and water.^[3,4]^ Experimental and epidemiologic evidence suggests that cadmium may contribute to oxidative stress, endothelial dysfunction, inflammation, and other atherosclerosis-related processes, supporting a possible link with cardiovascular disease.^[3,4]^ Consistent with this, systematic reviews and population-based studies have associated cadmium exposure with adverse cardiovascular outcomes; however, much of the existing literature has focused on overall cardiovascular disease, mortality, or non-CHD endpoints, and evidence specifically addressing prevalent CHD remains limited.^[4,5]^ Additional analyses in nationally representative adult populations are therefore warranted, particularly when smoking-related confounding is considered carefully.

Machine-learning approaches may complement conventional regression by capturing nonlinear patterns and interaction structures that may improve risk discrimination, but they do not replace etiologic inference and require careful attention to transparency, calibration, and validation.^[6–8]^ Accordingly, in this cross-sectional study, reported in accordance with the Strengthening the Reporting of Observational Studies in Epidemiology statement and based on data from the National Health and Nutrition Examination Survey (NHANES) 2013 to 2014, we pursued 2 related aims. First, we examined the association between blood cadmium concentrations and prevalent CHD in US adults using survey-weighted logistic regression. Second, we evaluated the discriminative performance of multiple machine-learning models and examined the importance of blood cadmium in the final model using Shapley Additive Explanations (SHAP). We considered the machine-learning analyses exploratory and used them to complement, rather than replace, the regression-based association analyses.

## 2. Methods

### 2.1. Study design and study population

This cross-sectional study was reported in accordance with the Strengthening the Reporting of Observational Studies in Epidemiology statement and used publicly available data from NHANES 2013 to 2014, a continuous program designed to generate nationally representative estimates for the noninstitutionalized US civilian population through a complex, multistage probability sampling design.^[9,10]^ The NHANES protocol was approved by the National Center for Health Statistics Ethics Review Board, and all participants provided written informed consent before participation.^[10]^

**Figure 1.**
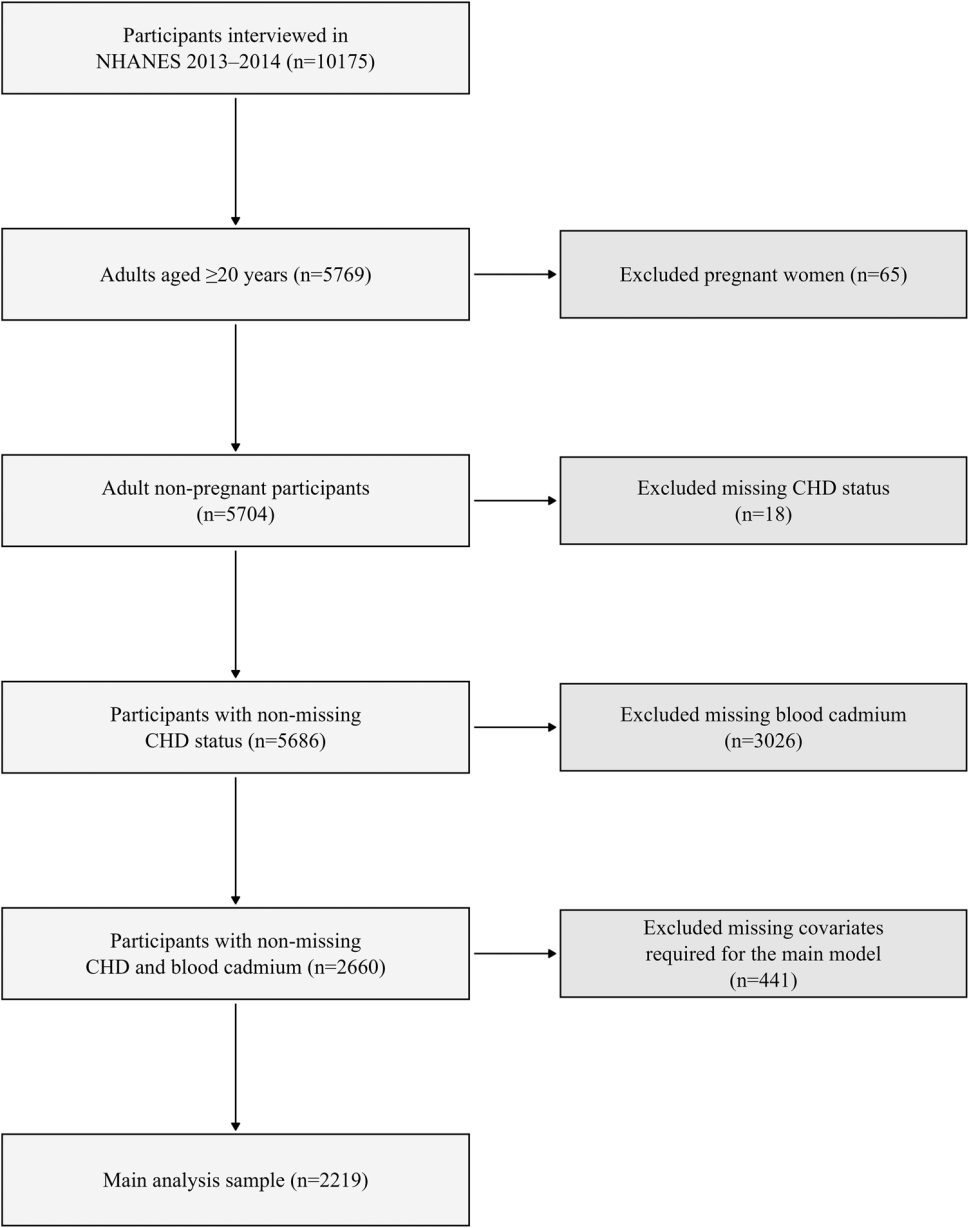
Flowchart of participant selection from NHANES 2013 to 2014. Adults aged 20 years or older were identified from NHANES 2013 to 2014. Pregnant participants, those with missing CHD status, those without blood cadmium measurements, and those with missing covariates required for the fully adjusted model were excluded. The final complete-case analytic sample included 2219 participants. CHD = coronary heart disease, NHANES = National Health and Nutrition Examination Survey.

A total of 10,175 participants were interviewed in NHANES 2013 to 2014. We first identified 5769 adults aged 20 years or older. We then excluded pregnant women (n = 65), participants with missing CHD status (n = 18), participants without blood cadmium measurements (n = 3026), and participants with missing covariates required for the fully adjusted main model (n = 441). The final complete-case analytic sample included 2219 participants (Fig. 1). Because the primary analysis was based on complete-case data, we additionally compared included and excluded adult nonpregnant participants to evaluate the potential for selection bias.

### 2.2. Assessment of blood cadmium and prevalent CHD

Whole-blood cadmium was the exposure of interest. In NHANES 2013 to 2014, blood cadmium was measured by inductively coupled plasma mass spectrometry according to the National Center for Environmental Health laboratory protocol.^[11]^ The lower limit of detection (LLOD) for blood cadmium was 0.10 μg/L.^[11]^ For values below the LLOD, NHANES assigned an imputed value equal to the LLOD divided by the square root of 2.^[11]^ Blood cadmium was analyzed both as a continuous variable after natural logarithmic transformation and as quartiles based on the empirical distribution in the analytic sample: Q1: ≤0.170 μg/L; Q2: 0.170–0.270 μg/L; Q3: 0.270–0.490 μg/L; and Q4: >0.490 μg/L.

The primary outcome was prevalent CHD, defined by a positive response to the NHANES medical conditions questionnaire item asking whether a doctor or other health professional had ever told the participant that he or she had coronary heart disease. Participants answering “yes” were classified as having prevalent CHD, whereas those answering “no” were classified as not having prevalent CHD.

### 2.3. Covariates

Covariates for the survey-weighted regression analyses were selected a priori on the basis of clinical relevance and their potential roles as confounders of the association between blood cadmium and prevalent CHD. These covariates included age, sex, race/ethnicity, education level, poverty income ratio, body mass index (BMI), hypertension, alcohol use, smoking status, total cholesterol, and high-density lipoprotein (HDL) cholesterol. Marital status was additionally included in the machine-learning feature set.

Race/ethnicity was categorized as Mexican American, other Hispanic, non-Hispanic White, non-Hispanic Black, and other race. Education level was categorized as below 9th grade, 9th to 11th grade, high school graduate, some college, and college or above. BMI was calculated as weight in kilograms divided by height in meters squared. Hypertension was defined as systolic blood pressure ≥140 mm Hg, diastolic blood pressure ≥90 mm Hg, self-reported physician-diagnosed hypertension, or current use of antihypertensive medication.

Smoking status was categorized as never, former, or current using NHANES questionnaire data on lifetime cigarette exposure and current smoking behavior.^[12]^ Because smoking is a major source of cadmium exposure, serum cotinine was additionally considered an objective marker of tobacco exposure and was incorporated in sensitivity analyses. Alcohol use was coded dichotomously from questionnaire data.

### 2.4. Survey-weighted regression analyses and sensitivity analyses

All descriptive and regression analyses accounted for the NHANES complex sampling design using the 2-year blood metal subsample weights, strata, and primary sampling units, in accordance with NHANES analytic guidance for laboratory subsamples.^[10,11]^ Continuous variables are presented as mean ± standard deviation or median (interquartile range), as appropriate, and categorical variables are presented as weighted n (%). Differences across blood cadmium quartiles were evaluated using weighted linear regression for continuous variables and weighted chi-square tests for categorical variables.

We used survey-weighted logistic regression to estimate odds ratios (ORs) and 95% confidence intervals (CIs) for prevalent CHD in relation to blood cadmium. Blood cadmium was modeled both as a natural log-transformed continuous variable and as quartiles, with the lowest quartile as the reference group. Three models were fitted: a crude model with no covariate adjustment; Model 1 adjusted for age, sex, and race/ethnicity; and Model 2 additionally adjusted for education level, poverty income ratio, BMI, hypertension, alcohol use, smoking status, total cholesterol, and HDL cholesterol. Tests for trend across quartiles were performed by entering quartile rank as an ordinal variable.

No multivariable imputation was used in the primary analysis. Instead, the main regression models were fitted in participants with complete data for prevalent CHD, blood cadmium, and all covariates required for the fully adjusted model. To evaluate the potential impact of exclusions, we compared included and excluded adult nonpregnant participants on key baseline characteristics.

Several sensitivity analyses were prespecified to address smoking-related confounding. First, we repeated the fully adjusted model after additional adjustment for serum cotinine. Second, we repeated the fully adjusted model among self-reported never smokers only. Third, we performed a stricter never-smoker analysis restricted to participants who both self-reported never smoking and had serum cotinine concentrations <10 ng/mL. These analyses were intended to assess the robustness of the association between blood cadmium and prevalent CHD to residual confounding by tobacco exposure.

### 2.5. Machine-learning model development and Bayesian hyperparameter optimization

To complement the association analyses, we developed supervised machine-learning models to evaluate the discriminative performance of a limited set of candidate features for prevalent CHD. The machine-learning component was exploratory and was intended to assess predictive discrimination and model interpretability rather than support etiologic or population-level inference. Because most candidate machine-learning algorithms do not directly accommodate the NHANES complex survey design, survey weights were not used in the machine-learning analyses. Therefore, population-level inference relied on the survey-weighted logistic regression models. Given the limited number of CHD events, the machine-learning analyses were restricted to a parsimonious set of candidate variables. Feature screening was performed within the training set in 2 stages, first by univariable analyses and then by multivariable analyses, before specification of the final feature set.

The complete analytic dataset was randomly divided into a training set (70%) and a testing set (30%) using stratified sampling according to CHD status. The final machine-learning feature set comprised 8 variables: age, blood cadmium, sex, race/ethnicity, education level, marital status, smoking status, and hypertension. Continuous predictors were standardized using parameters estimated from the training set, and categorical predictors were encoded within the modeling pipeline to avoid information leakage.

Ten supervised machine-learning algorithms were developed: logistic regression, support vector machine, k-nearest neighbors (KNN), multilayer perceptron (MLP), random forest, gradient boosting decision tree, AdaBoost, extreme gradient boosting, light gradient boosting machine, and CatBoost. Hyperparameters were optimized via Bayesian tuning implemented with the Optuna framework and 5-fold cross-validation within the training set.^[13]^ The primary optimization target was mean cross-validated area under the receiver operating characteristic curve (AUROC). For each algorithm, the final optimized hyperparameters were retained, and the model was refitted using the full training set. Detailed hyperparameter search settings and the final optimized values for all models are provided in Supplementary Table 2, Supplemental Digital Content 1.

Model performance was evaluated in the held-out test set using the AUROC, accuracy, sensitivity, specificity, positive predictive value, and negative predictive value (NPV). In addition to discrimination, overall prediction error and calibration were assessed to avoid overreliance on discrimination alone.^[14,15]^ The best-performing model was identified on the basis of a balanced assessment of discrimination, calibration, and classification performance.

### 2.6. Calibration assessment, model interpretability, and software

Calibration was assessed using the Brier score, calibration intercept, and calibration slope, rather than relying on simple goodness-of-fit testing alone, in line with contemporary recommendations for prediction model evaluation.^[14,15]^ Calibration curves were constructed by grouping test-set predicted probabilities into quantile-based bins and plotting the observed event proportion against the mean predicted probability within each bin. No post hoc recalibration procedure was applied before evaluation.

For the best-performing final model, we used SHAP to quantify model-based feature importance and to visualize the direction and magnitude of feature contributions across individuals.^[16]^ Global feature importance was summarized using the mean absolute SHAP value for each feature, and individual-level contribution patterns were visualized using a SHAP feature-importance bar plot and a SHAP beeswarm plot.

To further characterize the role of blood cadmium in the final model, we generated a partial dependence plot (PDP) for blood cadmium.^[17]^ The PDP was used to display the marginal association between blood cadmium and the model-predicted probability of prevalent CHD while averaging over the observed distribution of the remaining model features. Any apparent change in slope on the PDP was interpreted as a descriptive feature of the fitted model rather than a clinical threshold.

All survey-weighted analyses were performed in R (version 4.2.0). Machine-learning analyses were conducted in Python (version 3.9) using a prespecified modeling workflow adapted for tabular clinical data, including Bayesian hyperparameter optimization and model interpretability analyses. All statistical tests were 2-sided, and *P* < .05 was considered statistically significant.

## 3. Results

### 3.1. Participant characteristics

A total of 2219 adults were included in the main analytic sample, among whom 80 had prevalent CHD. The weighted baseline characteristics of the study population according to quartiles of blood cadmium are shown in Table 1. Participants in higher blood cadmium quartiles tended to be older and were more likely to have hypertension and current smoking. Poverty income ratio also differed across quartiles and was lowest in the highest quartile (Q4). Smoking-related gradients across quartiles were particularly marked: the proportion of current smokers increased from 1.7% in the lowest quartile (Q1) to 55.4% in Q4, and median serum cotinine concentrations also increased substantially across quartiles. Prevalent CHD differed significantly across quartiles, increasing from 1.2% in Q1 to 5.6% in Q4 (*P* = .014).

**Table 1:** Baseline characteristics of study participants according to quartiles of blood cadmium concentrations.

Characteristic	Overall N = 195,384,227*	Q1 (≤0.170) N = 54,908,300*	Q2 (0.170–0.270) N = 44,546,599*	Q3 (0.270–0.490) N = 47,507,936*	Q4 (>0.490) N = 48,421,392*	*P*-value†
Age (yr)	47.77 ± 17.05	40.72 ± 15.43	48.46 ± 16.52	52.89 ± 17.08	50.10 ± 16.70	<.001
Gender						<.001
Male	97,497,725 (49.9%)	35,473,718 (64.6%)	24,651,383 (55.3%)	18,438,321 (38.8%)	18,934,302 (39.1%)	
Female	97,886,501 (50.1%)	19,434,582 (35.4%)	19,895,215 (44.7%)	29,069,615 (61.2%)	29,487,089 (60.9%)	
Race						.001
Mexican American	16,466,432 (8.4%)	5134,108 (9.4%)	5679,088 (12.7%)	3303,087 (7.0%)	2350,149 (4.9%)	
Other Hispanic	9960,980 (5.1%)	3199,305 (5.8%)	2289,154 (5.1%)	2319,899 (4.9%)	2152,623 (4.4%)	
Non-Hispanic White	133,100,934 (68.1%)	38,709,444 (70.5%)	29,170,395 (65.5%)	32,473,774 (68.4%)	32,747,321 (67.6%)	
Non-Hispanic Black	20,674,520 (10.6%)	5361,010 (9.8%)	4472,713 (10.0%)	4689,650 (9.9%)	6151,146 (12.7%)	
Other Race	15,181,361 (7.8%)	2,504,433 (4.6%)	2,935,250 (6.6%)	4,721,527 (9.9%)	5,020,152 (10.4%)	
Education						<.001
<9th grade	8389,560 (4.3%)	2,639,528 (4.8%)	2,011,358 (4.5%)	1675,713 (3.5%)	2062,961 (4.3%)	
9–11th grade	18,315,527 (9.4%)	3,730,087 (6.8%)	2,538,095 (5.7%)	4,019,318 (8.5%)	8,028,027 (16.6%)	
High school	43,702,010 (22.4%)	11,903,187 (21.7%)	11,434,667 (25.7%)	7564,424 (15.9%)	12,799,732 (26.4%)	
Some college	63,652,380 (32.6%)	18,276,307 (33.3%)	13,727,067 (30.8%)	15,476,649 (32.6%)	16,172,357 (33.4%)	
College or above	61,324,750 (31.4%)	18,359,192 (33.4%)	14,835,412 (33.3%)	18,771,831 (39.5%)	9,358,315 (19.3%)	
Marital status						<.001
Married	107,354,846 (54.9%)	30,790,594 (56.1%)	28,356,893 (63.7%)	26,943,023 (56.7%)	21,264,336 (43.9%)	
Living with partner	13,535,463 (6.9%)	3028,558 (5.5%)	2395,758 (5.4%)	2885,931 (6.1%)	5225,216 (10.8%)	
Never married	36,846,611 (18.9%)	15,354,939 (28.0%)	6696,590 (15.0%)	6717,383 (14.1%)	8,077,698 (16.7%)	
Divorced	22,412,482 (11.5%)	4027,043 (7.3%)	4705,783 (10.6%)	5687,009 (12.0%)	7992,646 (16.5%)	
Separated	3,889,428 (2.0%)	828,817 (1.5%)	600,171 (1.3%)	600,708 (1.3%)	1859,731 (3.8%)	
Widowed	11,345,398 (5.8%)	878,348 (1.6%)	1791,403 (4.0%)	4673,883 (9.8%)	4001,764 (8.3%)	
Poverty income ratio	2.94 ± 1.64	3.04 ± 1.63	3.18 ± 1.62	3.20 ± 1.60	2.34 ± 1.58	<.001
Body mass index (kg/m^2^)	29.13 ± 7.14	29.50 ± 7.22	29.72 ± 6.98	29.25 ± 7.11	28.05 ± 7.14	.008
SBP (mm Hg)	122.04 ± 17.16	119.95 ± 15.85	122.72 ± 16.51	122.61 ± 16.95	123.24 ± 19.13	.194
DBP (mm Hg)	69.89 ± 12.20	70.14 ± 10.79	70.89 ± 12.29	69.22 ± 12.66	69.33 ± 13.09	.197
Total cholesterol (mg/dL)	191.10 ± 40.38	186.74 ± 37.88	190.89 ± 40.21	193.24 ± 39.97	194.15 ± 43.25	.108
HDL cholesterol (mg/dL)	53.52 ± 16.79	50.82 ± 15.48	52.17 ± 15.87	56.16 ± 17.31	55.22 ± 17.93	<.001
Hypertension						.025
No	116,599,440 (59.7%)	35,543,945 (64.7%)	27,084,464 (60.8%)	27,731,879 (58.4%)	26,239,152 (54.2%)	
Yes	78,784,787 (40.3%)	19,364,355 (35.3%)	17,462,135 (39.2%)	19,776,058 (41.6%)	22,182,240 (45.8%)	
Alcohol use						.416
No	42,515,005 (21.8%)	11,408,360 (20.8%)	10,180,045 (22.9%)	11,511,036 (24.2%)	9415,564 (19.4%)	
Yes	152,869,222 (78.2%)	43,499,940 (79.2%)	34,366,554 (77.1%)	35,996,900 (75.8%)	39,005,828 (80.6%)	
Smoking status						<.001
Never	109,971,650 (56.3%)	45,230,558 (82.4%)	29,387,893 (66.0%)	25,686,463 (54.1%)	9666,736 (20.0%)	
Former	49,602,107 (25.4%)	8761,785 (16.0%)	13,127,311 (29.5%)	15,759,644 (33.2%)	11,953,367 (24.7%)	
Current	35,810,470 (18.3%)	915,957 (1.7%)	2031,395 (4.6%)	6,061,830 (12.8%)	26,801,288 (55.4%)	
Serum cotinine, ng/mL	0.03 (0.01, 7.54)	0.02 (0.01, 0.08)	0.02 (0.01, 0.10)	0.02 (0.01, 0.23)	147.00 (0.04, 287.00)	<.001
Coronary heart disease	6,088,462 (3.1%)	640,120 (1.2%)	1,222,097 (2.7%)	1,501,636 (3.2%)	2724,610 (5.6%)	.014

*Note*: Continuous variables are presented as mean ± standard deviation or median (interquartile range), as appropriate, and categorical variables are presented as weighted n (%). Group differences in continuous variables were assessed using survey-weighted linear regression, and group differences in categorical variables were assessed using the weighted chi-square test. Quartiles of blood cadmium were defined as Q1 (≤0.170 μg/L), Q2 (0.170–0.270 μg/L), Q3 (0.270–0.490 μg/L), and Q4 (>0.490 μg/L).

CHD = coronary heart disease, DBP = diastolic blood pressure, HDL = high-density lipoprotein, SBP = systolic blood pressure.

* Weighted population estimate.

† *P* values were calculated using survey-weighted linear regression for continuous variables and the weighted chi-square test for categorical variables.

### 3.2. Association between blood cadmium and prevalent CHD

The associations between blood cadmium concentrations and prevalent CHD are presented in Table 2. In the crude model, each 1-unit increase in ln-transformed blood cadmium was associated with higher odds of prevalent CHD (OR, 1.65; 95% CI, 1.35–2.01; *P* = .001). This association remained after adjustment for age, sex, and race/ethnicity (Model 1: OR, 1.52; 95% CI, 1.20–1.92; *P* = .001) and after further adjustment for education, poverty income ratio, BMI, hypertension, alcohol use, smoking status, total cholesterol, and HDL cholesterol (Model 2: OR, 1.38; 95% CI, 1.05–1.81; *P* = .021).

**Table 2:** Associations of blood cadmium concentrations with prevalent coronary heart disease in survey-weighted logistic regression models.

		Crude model		Model 1		Model 2	
Exposure	Comparison	OR (95% CI)	*P*-value	OR (95% CI)	*P*-value	OR (95% CI)	*P*-value
Ln blood cadmium	Per 1-unit increase	1.65 (1.35–2.01)	.001	1.52 (1.20–1.92)	.001	1.38 (1.05–1.81)	.021
Blood cadmium quartiles	Q2 vs Q1	1.30 (0.95–1.78)	.102	1.25 (0.90–1.73)	.185	1.15 (0.82–1.61)	.415
	Q3 vs Q1	1.85 (1.30–2.63)	.001	1.60 (1.10–2.33)	.014	1.45 (1.02–2.06)	.039
	Q4 vs Q1	2.80 (1.90–4.12)	.001	2.30 (1.50–3.52)	<.001	1.95 (1.25–3.04)	.003
	*P* for trend		<.001		<.001		.002

*Note*: ORs and 95% CIs were estimated using survey-weighted logistic regression models. Blood cadmium was analyzed both as a continuous variable after natural log transformation and as quartiles. For the continuous analysis, ORs represent the association with prevalent CHD per 1-unit increase in ln-transformed blood cadmium concentration. For the categorical analysis, the lowest quartile (Q1) was used as the reference group. *P* for trend was calculated by modeling quartiles of blood cadmium as an ordinal variable. Crude model was unadjusted. Model 1 was adjusted for age, sex, and race/ethnicity. Model 2 was additionally adjusted for education, poverty income ratio, body mass index, hypertension, alcohol use, smoking status, total cholesterol, and HDL cholesterol. Quartiles of blood cadmium were defined according to the empirical quartile cut-points in the main analytic sample.

CHD = coronary heart disease, CI = confidence interval, HDL = high-density lipoprotein, OR = odds ratio.

When blood cadmium was modeled by quartiles, participants in the 3rd and 4th quartiles had higher odds of prevalent CHD than those in the lowest quartile in the fully adjusted model. Specifically, the OR was 1.45 (95% CI, 1.02–2.06; *P* = .039) for the third quartile (Q3) versus the lowest quartile (Q1) and 1.95 (95% CI, 1.25–3.04; *P* = .003) for Q4 versus Q1. A significant trend across quartiles remained evident in the fully adjusted model (*P* for trend = 0.002), consistent with a graded association between higher blood cadmium concentrations and prevalent CHD.

### 3.3. Sensitivity analyses and assessment of selection bias

The sensitivity analyses are summarized in Table 3. After additional adjustment for serum cotinine, the association between ln-transformed blood cadmium and prevalent CHD remained statistically significant (OR, 1.30; 95% CI, 1.01–1.67; *P* = .042). Likewise, participants in the highest quartile had higher odds of prevalent CHD than those in the lowest quartile (OR, 1.75; 95% CI, 1.12–2.73), and the trend across quartiles remained significant (*P* for trend = 0.015).

**Table 3:** Sensitivity analyses of the associations between blood cadmium concentrations and prevalent coronary heart disease.

Analysis	N	Cases	OR per ln-cadmium (95% CI)	*P*-value	Q4 vs Q1 OR (95% CI)	*P* for trend
Main analysis	2219	80	1.38 (1.05–1.81)	.021	1.95 (1.25–3.04)	.002
Additional adjustment for serum cotinine	2216	79	1.30 (1.01–1.67)	.042	1.75 (1.12–2.73)	.015
Self-reported never smokers	1258	31	1.22 (0.92–1.62)	.168	1.48 (0.95–2.30)	.085
Strict never smokers (self-report + cotinine < 10 ng/mL)	1195	31	1.20 (0.88–1.63)	.251	1.45 (0.91–2.31)	.120

*Note:* Sensitivity analyses were performed using survey-weighted logistic regression models. Blood cadmium was analyzed as a continuous variable after natural log transformation and as quartiles. ORs for ln-blood cadmium represent the association with prevalent CHD per 1-unit increase in ln-transformed blood cadmium concentration. For quartile analyses, the lowest quartile (Q1) was used as the reference group, and P for trend was calculated by modeling quartiles as an ordinal variable. The main analysis was adjusted for age, sex, race/ethnicity, education, poverty income ratio, body mass index, hypertension, alcohol use, smoking status, total cholesterol, and HDL cholesterol. The cotinine-adjusted analysis additionally included serum cotinine. N and Cases are unweighted counts in each analytic sample. Quartiles of blood cadmium were defined according to the empirical quartile cut-points in the main analytic sample.

CHD = coronary heart disease, CI = confidence interval, HDL = high-density lipoprotein, OR = odds ratio.

In analyses restricted to self-reported never smokers, the direction of association was preserved but was attenuated and no longer statistically significant (OR per 1-unit increase in ln-transformed blood cadmium, 1.22; 95% CI, 0.92–1.62; *P* = .168; OR for Q4 vs Q1, 1.48; 95% CI, 0.95–2.30; *P* for trend = 0.085). Similar attenuation was observed in the stricter never-smoker subgroup defined by self-reported never smoking and serum cotinine <10 ng/mL (OR per 1-unit increase, 1.20; 95% CI, 0.88–1.63; *P* = .251; OR for Q4 vs Q1, 1.45; 95% CI, 0.91–2.31; *P* for trend = 0.120).

To evaluate the potential for selection bias related to exclusions, we compared included and excluded participants in the adult nonpregnant NHANES 2013 to 2014 sample (Supplementary Table 3, Supplemental Digital Content 2). The 2 groups were generally similar with respect to age, sex, education, marital status, BMI, blood pressure, HDL cholesterol, hypertension, alcohol use, smoking status, serum cotinine, and prevalent CHD. Modest differences were observed for race/ethnicity, poverty income ratio, and total cholesterol. Importantly, prevalent CHD did not differ significantly between included and excluded participants (3.1% vs 4.0%, *P* = .210).

### 3.4. Machine-learning performance and calibration

**Figure 2.**
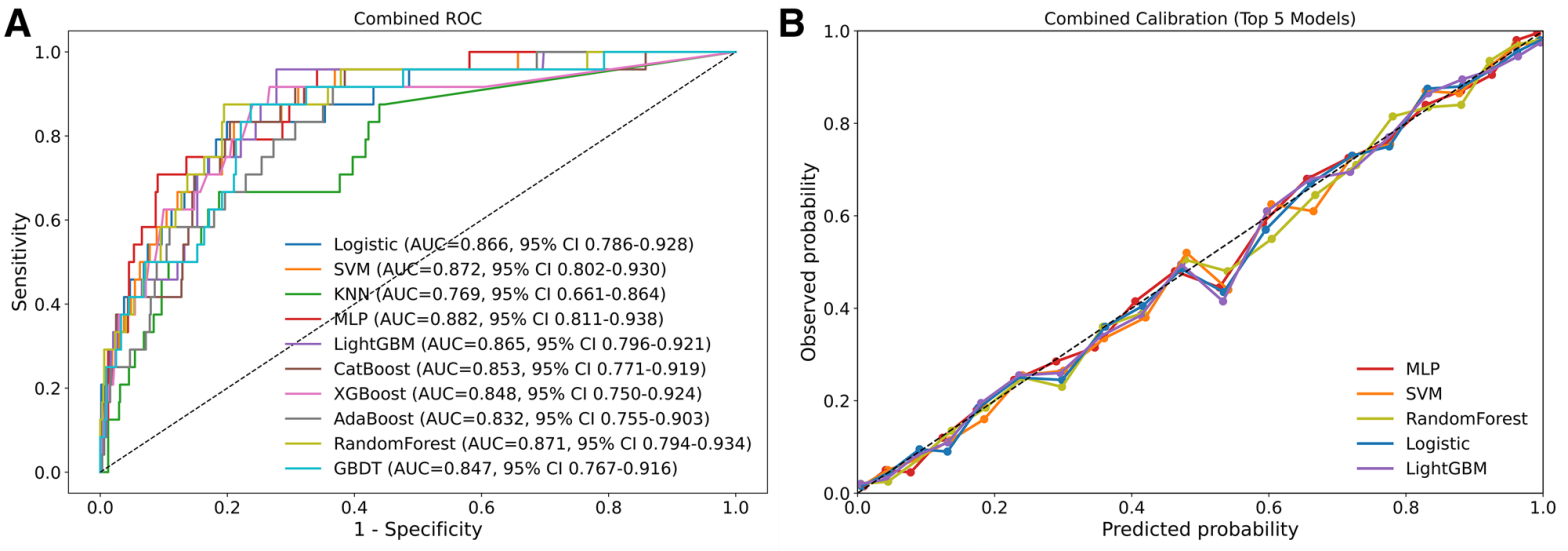
Receiver operating characteristic curves of the 10 machine-learning models for prevalent CHD. Receiver operating characteristic curves are shown for LR, SVM, KNN, MLP, RF, GBDT, AdaBoost, XGBoost, LightGBM, and CatBoost in the held-out test set. Among the evaluated models, the MLP achieved the highest AUROC and was selected as the final model for interpretability analyses. CHD = coronary heart disease, GBDT = gradient boosting decision tree, KNN = k-nearest neighbors, LightGBM = light gradient boosting machine, LR = logistic regression, MLP = multilayer perceptron, RF = random forest, SVM = support vector machine, XGBoost = extreme gradient boosting.

The performance and calibration metrics of the machine-learning models are summarized in Supplementary Table 1, Supplemental Digital Content 3, and the receiver operating characteristic (ROC) curves are shown in Figure 2. Among the 10 evaluated models, the MLP achieved the best overall discriminative performance, with an AUROC of 0.882 (95% CI, 0.811–0.938). The MLP also showed high accuracy (0.902; 95% CI, 0.879–0.923), specificity (0.911; 95% CI, 0.890–0.933), and NPV (0.987; 95% CI, 0.977–0.995), with a sensitivity of 0.667 (95% CI, 0.456–0.861) and a positive predictive value of 0.219 (95% CI, 0.125–0.324).

Several other models also demonstrated good discrimination, including support vector machine (AUROC, 0.872; 95% CI, 0.802–0.930), random forest (AUROC, 0.871; 95% CI, 0.794–0.934), and logistic regression (AUROC, 0.866; 95% CI, 0.786–0.928). By contrast, k-nearest neighbors showed the lowest AUROC among the evaluated models (0.769; 95% CI, 0.661–0.864). Light gradient boosting machine provided high sensitivity (0.917; 95% CI, 0.789–1.000) and very high NPV (0.996; 95% CI, 0.989–1.000), but with lower overall accuracy than the MLP.

Calibration results also favored the MLP. The MLP had a Brier score of 0.031 (95% CI, 0.020–0.043), a calibration intercept of −0.007, and a calibration slope of 0.963, indicating close agreement between predicted and observed probabilities. Its calibration curve also showed good agreement with the 45-degree reference line (Supplementary Figure 1, Supplemental Digital Content 4). Although several other models had similarly low Brier scores, calibration was less favorable for some algorithms, particularly AdaBoost, which showed a substantially higher Brier score (0.196; 95% CI, 0.193–0.198) and a lower calibration slope (0.505).

### 3.5. SHAP-based model interpretation and partial dependence analysis

**Figure 3.**
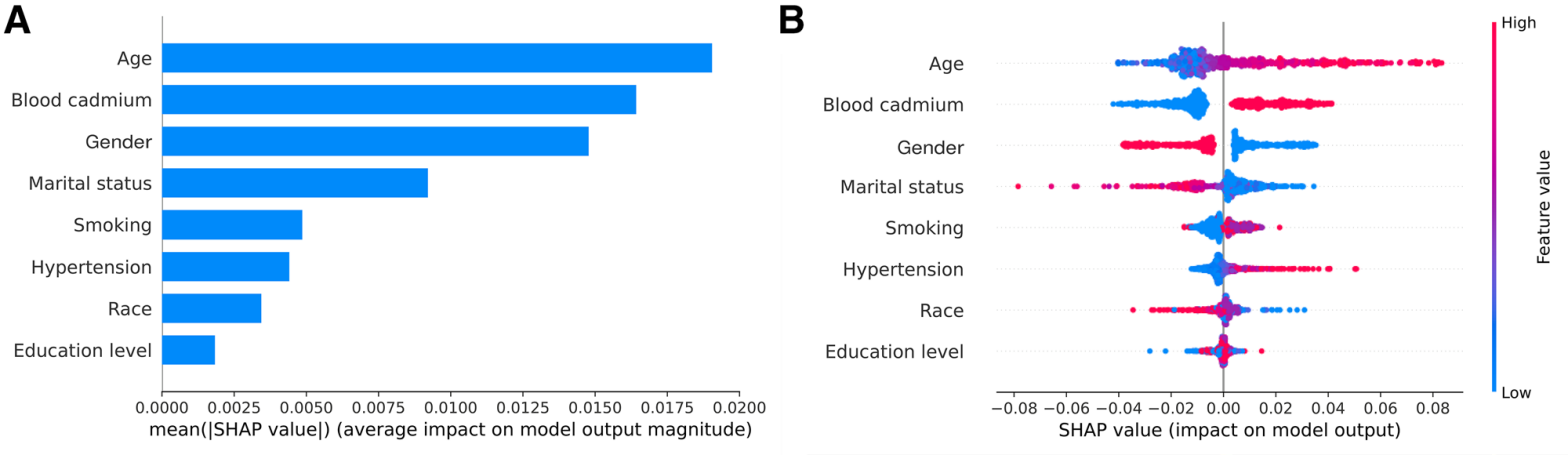
SHAP-based interpretation of the final MLP model for prevalent CHD. (A) Bar plot of global feature importance ranked by mean absolute SHAP value. Larger values indicate greater overall contribution of a feature to model output. (B) SHAP beeswarm plot showing the distribution of individual SHAP values for each feature. Each point represents 1 participant. Point color reflects the relative feature value from low to high, and the horizontal position reflects the direction and magnitude of the feature’s contribution to the model-predicted probability of prevalent CHD. CHD = coronary heart disease, MLP = multilayer perceptron, SHAP = Shapley Additive Explanations.

Because the MLP provided the best overall combination of discrimination and calibration, it was selected as the final model for interpretability analyses. SHAP-based global feature importance is shown in Figure 3A. Age had the highest mean absolute SHAP value, followed by blood cadmium, indicating that blood cadmium ranked among the most important features in the final model. Other influential features included smoking status, hypertension, and sex.

The SHAP beeswarm plot is shown in Figure 3B. Higher blood cadmium values were generally associated with positive SHAP values, suggesting a greater contribution to the model-predicted probability of prevalent CHD. However, some overlap between low and high feature values was present, suggesting that the contribution of blood cadmium varied across individuals within the broader multivariable context.

**Figure 4.**
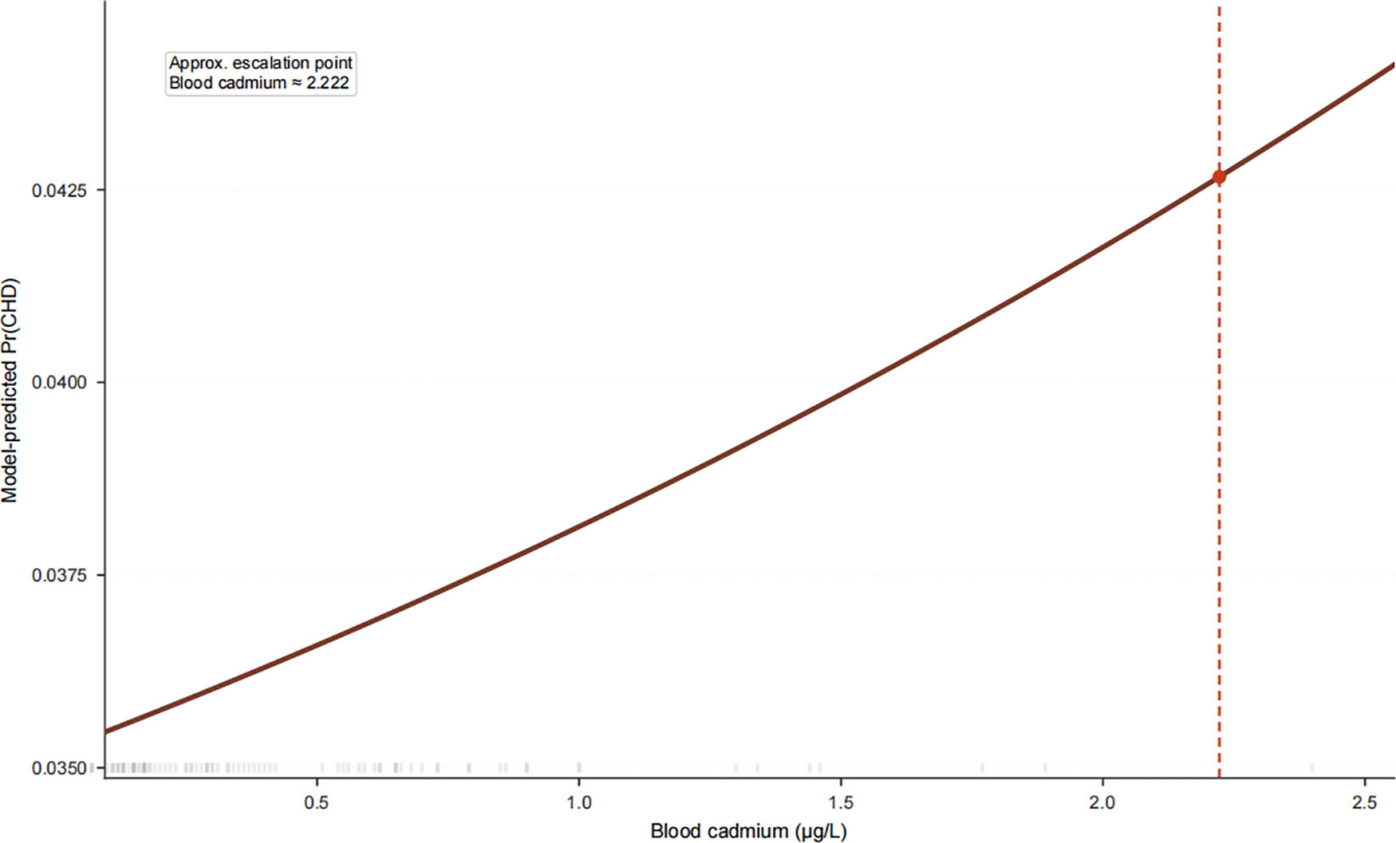
Partial dependence plot of blood cadmium in the final MLP model. The plot shows the marginal relationship between blood cadmium concentration and the model-predicted probability of prevalent CHD while averaging over the observed distribution of the remaining model features. Rug marks along the *x*-axis indicate the distribution of observed blood cadmium values. The dashed vertical line denotes the approximate point at which the fitted curve appeared to steepen (around 2.22 μg/L). Only 48 of 2219 participants (2.2%) had blood cadmium concentrations above this value, indicating that it lay in a relatively sparse upper-tail region of the observed distribution. This point should therefore be interpreted cautiously as a descriptive feature of the fitted model rather than a clinical threshold. CHD = coronary heart disease, MLP = multilayer perceptron.

Figure 4 shows the PDP for blood cadmium. The curve generally increased across the observed range of blood cadmium concentrations and appeared to steepen at around 2.22 μg/L. Only 48 of 2219 participants (2.2%) had blood cadmium concentrations above this value, indicating that it lay in a relatively sparse upper-tail region of the observed distribution. Given the cross-sectional and exploratory nature of this analysis, this value was interpreted as a descriptive feature of the fitted model rather than a clinical threshold.

## 4. Discussion

In this nationally representative cross-sectional study of US adults from NHANES 2013 to 2014, higher blood cadmium concentrations were associated with higher odds of prevalent CHD. This association was observed when blood cadmium was modeled both as a natural log-transformed continuous variable and by quartiles, and a significant trend across quartiles remained after adjustment for major demographic, socioeconomic, behavioral, and clinical covariates. In the machine-learninganalyses, the final MLP model showed the best overall combination of discrimination and calibration in this dataset, and blood cadmium ranked among the most important model features. Taken together, these findings support an association between higher blood cadmium concentrations and prevalent CHD and suggest that blood cadmium also contributed to predictive performance in the final model.

Our findings are broadly consistent with prior reviews and meta-analyses linking cadmium exposure to adverse cardiovascular outcomes, including CHD.^[3–5]^ Importantly, the magnitude of association observed in our study is also directionally consistent with prospective estimates reported in original cohort studies, although direct numerical comparison should be made cautiously because of differences in exposure biomarkers, outcome definitions, and analytic contrasts. In our fully adjusted model, the OR for prevalent CHD was 1.95 when comparing the highest with the lowest blood cadmium quartile. In a prospective analysis of NHANES 1999 to 2004, Tellez-Plaza et al reported a hazard ratio of 1.73 for CHD mortality when comparing the 80th with the 20th percentile of blood cadmium.^[18]^ In the Strong Heart Study, the same group also reported a hazard ratio of 1.22 for incident CHD and 1.34 for CHD mortality when comparing corresponding percentiles of urine cadmium.^[19]^ Although these studies are not directly comparable to our cross-sectional analysis of prevalent CHD, they place our findings within a broader evidence base, suggesting that cadmium exposure is consistently associated with coronary outcomes. In this context, the present analysis adds several elements to the literature. First, it specifically addresses prevalent CHD in a contemporary NHANES cycle rather than broader composite cardiovascular outcomes. Second, it combines survey-weighted association analyses with machine-learning discrimination and interpretability analyses. Third, it incorporates additional sensitivity analyses aimed at addressing smoking-related confounding, which remains a central challenge in cadmium epidemiology.^[3,20]^

Smoking deserves particular emphasis because cigarette smoke is a major source of cadmium exposure, and in our data, smoking-related gradients across blood cadmium quartiles were substantial.^[20,21]^ This makes residual confounding by tobacco exposure a legitimate concern even when smoking status is included as a covariate. In our study, the association between blood cadmium and prevalent CHD remained statistically significant after additional adjustment for serum cotinine, suggesting that the observed association was not fully accounted for by smoking-status classification alone. At the same time, the attenuation of the association in the never-smoker and strict never-smoker subgroup analyses argues for caution. These subgroup results may reflect residual confounding, reduced statistical power, narrower exposure variability, or a combination of these factors. Therefore, although cadmium is biologically toxic, our data do not establish cadmium from nonsmoking environmental sources, such as food or water, as an independent driver of CHD. A more defensible interpretation is that higher blood cadmium concentrations were associated with prevalent CHD in the overall sample, whereas the magnitude and independence of that association remain sensitive to how tobacco exposure is handled analytically.

The observed association is biologically plausible. Contemporary reviews and the American Heart Association scientific statement describe several pathways through which cadmium may contribute to cardiovascular injury, including oxidative stress, endothelial dysfunction, chronic inflammation, dyslipidemia, plaque instability, and thrombosis-related processes.^[3,20]^ These mechanisms are compatible with an atherosclerotic framework and may help explain why cadmium has been repeatedly linked to ischemic cardiovascular outcomes in population-based studies.^[3–5]^ However, biological plausibility should not be conflated with proof of causality in the present study. Because our analysis was cross-sectional and relied on self-reported prevalent CHD, reverse causation and residual confounding cannot be excluded.

The machine-learning results require similarly careful interpretation. In the final MLP model, age had the highest mean absolute SHAP value, and blood cadmium ranked second, indicating that blood cadmium was among the most important features for model output in this dataset. This does not imply that cadmium is more etiologically important than hypertension or other established cardiovascular risk factors. SHAP importance is model-specific and reflects the contribution of a feature within the fitted multivariable prediction structure, which can be influenced by feature distributions, correlation patterns, nonlinearities, and interactions. The overlap in the SHAP beeswarm plot further suggests that the contribution of blood cadmium varied across individuals and was influenced by the broader multivariable context. Likewise, the PDP appeared to steepen at around 2.22 μg/L, but only 48 of 2219 participants (2.2%) had blood cadmium concentrations above this value. This indicates that the apparent steepening occurred in a relatively sparse upper-tail region and should therefore be interpreted cautiously. The pattern reflects the behavior of the fitted model and should not be used to define a clinical cut-point.

Despite these limitations, the machine-learning analyses provide complementary information when interpreted cautiously. The MLP achieved the best overall balance of discrimination and calibration in the held-out test set, and calibration was assessed using the Brier score, calibration intercept, calibration slope, and calibration curves, rather than relying only on a simple goodness-of-fit test.^[14,15]^ Our finding that blood cadmium contributed to predictive performance is also broadly consistent with prior predictive modeling work using NHANES data. In particular, Wang et al reported that adding blood metals to cardiovascular mortality prediction models improved the C-statistic in the testing set from 0.845 to 0.854, and to 0.857 when linear, squared, and pairwise interaction terms for blood metals were incorporated.^[22]^ Although that study focused on cardiovascular mortality rather than prevalent CHD and used a different modeling framework, it supports the broader notion that blood metal biomarkers may provide incremental prognostic information beyond conventional cardiovascular risk factors. Even so, the role of machine learning in this study was exploratory. The purpose of these models was to examine discriminative performance and relative feature importance within a limited tabular dataset, not to replace survey-weighted regression for association estimation or to support clinical deployment. Recent reviews of cardiovascular prediction models have emphasized that machine-learning performance is often dataset-specific and that translation to practice requires transparent reporting, calibration assessment, external validation, and careful consideration of clinical utility.^[6–8]^ Accordingly, our machine-learning findings should be viewed as complementary to, rather than stronger than, the survey-weighted regression results.

From a clinical and public health perspective, the present findings do not justify immediate changes in clinical practice, routine cadmium screening, or the use of a model-derived cadmium threshold for decision-making. Rather, they support growing concern that environmental metal exposures may contribute to the burden of coronary disease and should be considered alongside conventional cardiovascular prevention strategies, particularly smoking prevention and cessation and broader exposure-reduction efforts.^[3,20,21]^ Accordingly, these findings are more relevant to risk awareness and hypothesis generation than to immediate clinical decision-making.

### 4.1. Limitations

Several limitations should be acknowledged. First, the cross-sectional design precludes temporal inference; therefore, we cannot determine whether higher blood cadmium concentrations preceded CHD or were correlated with other factors related to prevalent disease. Second, the outcome was based on self-reported physician diagnosis of CHD, which may be subject to recall bias, outcome misclassification, and under-ascertainment of subclinical disease. Third, although we refined smoking adjustment and added serum cotinine and never-smoker sensitivity analyses, residual confounding by tobacco exposure remains possible. Fourth, the primary analysis used a complete-case design because blood cadmium and covariate missingness excluded a substantial number of participants. Although included and excluded participants were broadly similar for many characteristics, selection bias cannot be ruled out. Fifth, the machine-learning analyses did not incorporate the NHANES complex survey weights and therefore should not be interpreted as providing population-representative estimates. Finally, the interpretability analyses were based on a single final model without external validation, so the ranking and shape of feature contributions may not generalize to other populations or settings.

### 4.2. Future directions

Future studies should prioritize prospective designs with adjudicated coronary outcomes to clarify temporality and reduce outcome misclassification. Repeated assessment of blood and urinary cadmium may also help distinguish shorter-term exposure patterns from cumulative body burden. More refined tobacco exposure modeling, including pack-years, passive smoke exposure, and biomarker-informed approaches, will be important for disentangling cadmium-associated signals from smoking-related confounding. From a modeling perspective, external validation in independent populations, formal assessment of clinical utility, and, where feasible, development of survey-aware machine-learning strategies would improve the translational relevance of these findings. Additional work is also needed to determine whether cadmium improves risk stratification beyond established cardiovascular factors in clinically meaningful ways.

## 5. Conclusion

In this cross-sectional analysis of US adults from NHANES 2013 to 2014, higher blood cadmium concentrations were associated with higher odds of prevalent CHD. This association remained after multivariable adjustment and after additional adjustment for serum cotinine, but was attenuated in never-smoker subgroup analyses, supporting cautious interpretation in light of possible smoking-related confounding. Exploratory machine-learning analyses further suggested that blood cadmium was an important model feature in the final MLP model. Overall, these findings support an association between blood cadmium and prevalent CHD while underscoring the need for prospective studies with objective coronary outcomes and external validation before any clinical application is considered.

## Acknowledgments

The authors would like to express their gratitude to the NHANES team for providing the publicly available dataset used in this study. We also acknowledge the support of the Second Affiliated Hospital of Nanjing Medical University for facilitating this research.

## Author contributions

**Formal analysis:** Yi Li.

**Methodology:** Meng Liu.

**Software:** Ye Zhou.

**Writing – original draft: Hui Guo.**

**Writing – review & editing:** Mei Hong.








