# Supplementary material for: Association between blood cadmium and prevalent coronary heart disease in NHANES 2013 to 2014: A cross-sectional study with machine-learning analyses
Source: Medicine (Baltimore). 2026 Jul 3;105(27):e49554. doi: 10.1097/MD.0000000000049554 (PMC13337058; doi:10.1097/MD.0000000000049554)
Supplement: Supplementary file 1 [file medi-105-e49554-s001.docx]

## **Supplementary Table 2**. **Optimal hyperparameters of machine-learning models for predicting coronary heart disease.**

| Model | Hyperparameter | Value |
| --- | --- | --- |
| Logistic | penalty | l2 |
|  | C | 0.0047 |
|  | solver | lbfgs |
|  | max_iter | 2000 |
|  | random_state | 52 |
| SVM | gamma | auto |
|  | kernel | linear |
|  | max_iter | 3171 |
|  | C | 0.1774 |
| KNN | metric | minkowski |
|  | n_neighbors | 29 |
|  | weights | distance |
|  | p | 1 |
| MLP | activation | tanh |
|  | alpha | 0.0005 |
|  | learning_rate_init | 0.0051 |
|  | hidden_layer_sizes | 102, 38 |
| LightGBM | n_estimators | 854 |
|  | num_leaves | 40 |
|  | max_depth | 6 |
|  | min_child_samples | 5 |
|  | min_child_weight | 9.5549 |
|  | min_split_gain | 0.018 |
|  | subsample | 0.929 |
|  | bagging_fraction | 0.6147 |
|  | bagging_freq | 7 |
|  | colsample_bytree | 0.9177 |
|  | max_bin | 115 |
|  | learning_rate | 0.0145 |
|  | reg_lambda | 2.6415 |
|  | objective | binary |
|  | metric | auc |
|  | random_state | 52 |
|  | verbosity | -1 |
|  | force_col_wise | TRUE |
| CatBoost | iterations | 1828 |
|  | depth | 9 |
|  | subsample | 0.7866 |
|  | learning_rate | 0.0012 |
|  | l2_leaf_reg | 8.667 |
|  | loss_function | Logloss |
|  | eval_metric | AUC |
|  | random_seed | 52 |
|  | verbose | FALSE |
|  | allow_writing_files | FALSE |
| XGBoost | n_estimators | 109 |
|  | max_depth | 7 |
|  | subsample | 0.6067 |
|  | colsample_bytree | 0.8289 |
|  | min_child_weight | 2.7957 |
|  | gamma | 2.7721 |
|  | learning_rate | 0.0011 |
|  | reg_lambda | 0.0507 |
|  | reg_alpha | 0.9745 |
|  | objective | binary:logistic |
|  | eval_metric | auc |
|  | random_state | 52 |
|  | tree_method | hist |
|  | n_jobs | -1 |
| AdaBoost | n_estimators | 467 |
|  | learning_rate | 1.494 |
| RandomForest | n_estimators | 550 |
|  | max_depth | 5 |
|  | min_samples_split | 17 |
|  | min_samples_leaf | 10 |
|  | max_features | sqrt |
|  | bootstrap | TRUE |
| GBDT | n_estimators | 820 |
|  | max_depth | 3 |
|  | min_samples_split | 11 |
|  | min_samples_leaf | 6 |
|  | subsample | 0.7698 |
|  | learning_rate | 0.0015 |

Abbreviations: AdaBoost, adaptive boosting; CatBoost, categorical boosting; GBDT, gradient boosting decision tree; KNN, k-nearest neighbors; LightGBM, light gradient boosting machine; Logistic, logistic regression; MLP, multilayer perceptron; RandomForest, random forest; SVM, support vector machine; XGBoost, extreme gradient boosting; AUC, area under the receiver operating characteristic curve.
