# Supplementary material for: Association between blood cadmium and prevalent coronary heart disease in NHANES 2013 to 2014: A cross-sectional study with machine-learning analyses
Source: Medicine (Baltimore). 2026 Jul 3;105(27):e49554. doi: 10.1097/MD.0000000000049554 (PMC13337058; doi:10.1097/MD.0000000000049554)
Supplement: Supplementary file 2 [file medi-105-e49554-s002.docx]

**Supplementary Table 3. Comparison of included and excluded participants in the main analytic sample.**

| **Characteristic** | **Included N = 195,384,227^*^** | **Excluded N = 143,777,685^*^** | ***P*-value**^†^ |
| --- | --- | --- | --- |
| **Age, years** | 47.77 ± 17.05 | 47.72 ± 16.86 | 0.760 |
| **Gender** |  |  | 0.098 |
| Male | 97,497,725 (49.9%) | 66,783,355 (46.4%) |  |
| Female | 97,886,501 (50.1%) | 76,994,329 (53.6%) |  |
| **Race** |  |  | 0.005 |
| Mexican American | 16,466,432 (8.4%) | 14,580,741 (10.1%) |  |
| Other Hispanic | 9,960,980 (5.1%) | 8,659,590 (6.0%) |  |
| Non-Hispanic White | 133,100,934 (68.1%) | 90,719,585 (63.1%) |  |
| Non-Hispanic Black | 20,674,520 (10.6%) | 17,448,301 (12.1%) |  |
| Other Race | 15,181,361 (7.8%) | 12,369,466 (8.6%) |  |
| **Education** |  |  | 0.105 |
| <9th grade | 8,389,560 (4.3%) | 7,669,310 (5.3%) |  |
| 9-11th grade | 18,315,527 (9.4%) | 16,928,679 (11.8%) |  |
| High school | 43,702,010 (22.4%) | 31,360,610 (21.8%) |  |
| Some college | 63,652,380 (32.6%) | 45,811,126 (31.9%) |  |
| College or above | 61,324,750 (31.4%) | 41,857,530 (29.1%) |  |
| **Marital status** |  |  | 0.419 |
| Married | 107,354,846 (54.9%) | 80,124,070 (55.8%) |  |
| Living with partner | 13,535,463 (6.9%) | 9,488,801 (6.6%) |  |
| Never married | 36,846,611 (18.9%) | 26,685,367 (18.6%) |  |
| Divorced | 22,412,482 (11.5%) | 14,642,841 (10.2%) |  |
| Separated | 3,889,428 (2.0%) | 4,033,057 (2.8%) |  |
| Widowed | 11,345,398 (5.8%) | 8,715,051 (6.1%) |  |
| **Poverty income ratio** | 2.94 ± 1.64 | 2.86 ± 1.69 | 0.049 |
| **Body mass index, kg/m^2** | 29.13 ± 7.14 | 29.20 ± 7.08 | 0.672 |
| **SBP, mmHg** | 122.04 ± 17.16 | 121.77 ± 16.92 | 0.393 |
| **DBP, mmHg** | 69.89 ± 12.20 | 69.64 ± 12.06 | 0.374 |
| **Total cholesterol, mg/dL** | 191.10 ± 40.38 | 187.98 ± 42.74 | 0.019 |
| **HDL cholesterol, mg/dL** | 53.52 ± 16.79 | 52.84 ± 15.95 | 0.458 |
| **Hypertension** |  |  | 0.791 |
| No | 116,599,440 (59.7%) | 86,331,996 (60.1%) |  |
| Yes | 78,784,787 (40.3%) | 57,332,226 (39.9%) |  |
| **Alcohol use** |  |  | 0.164 |
| No | 42,515,005 (21.8%) | 28,629,084 (23.8%) |  |
| Yes | 152,869,222 (78.2%) | 91,499,198 (76.2%) |  |
| **Smoking status** |  |  | 0.129 |
| Never | 109,971,650 (56.3%) | 81,091,234 (56.4%) |  |
| Former | 49,602,107 (25.4%) | 32,296,347 (22.5%) |  |
| Current | 35,810,470 (18.3%) | 30,344,902 (21.1%) |  |
| **Serum cotinine, ng/mL** | 0.03 (0.01, 7.54) | 0.03 (0.01, 15.70) | 0.598 |
| **Coronary heart disease** | 6,088,462 (3.1%) | 5,678,014 (4.0%) | 0.210 |

Abbreviations: SBP, systolic blood pressure; DBP, diastolic blood pressure; HDL, high-density lipoprotein; CHD, coronary heart disease.

Note: The source population for this analysis was the adult non-pregnant NHANES 2013-2014 sample. Included participants were those with complete data for CHD, blood cadmium, and all covariates required for the fully adjusted main model. Excluded participants were the remaining adult non-pregnant participants. Continuous variables are presented as mean ± standard deviation or median (interquartile range), as appropriate, and categorical variables are presented as weighted n (%). Group differences in continuous variables were assessed using survey-weighted linear regression, and group differences in categorical variables were assessed using the weighted chi-square test.

^*^Weighted population estimate.

^†^*P* values were calculated using survey-weighted linear regression for continuous variables and the weighted chi-square test for categorical variables.
