# Supplementary material for: Association between blood cadmium and prevalent coronary heart disease in NHANES 2013 to 2014: A cross-sectional study with machine-learning analyses
Source: Medicine (Baltimore). 2026 Jul 3;105(27):e49554. doi: 10.1097/MD.0000000000049554 (PMC13337058; doi:10.1097/MD.0000000000049554)
Supplement: Supplementary file 3 [file medi-105-e49554-s003.docx]

Supplementary Table 1. Performance and calibration of machine-learning models for predicting coronary heart disease.

| **Model** | **Threshold** | **AUROC (95% CI)** | **Accuracy (95% CI)** | **Sensitivity (95% CI)** | **Specificity (95% CI)** | **PPV (95% CI)** | **NPV (95% CI)** | **Brier score (95% CI)** | **Calibration intercept** | **Calibration slope** |
| --- | --- | --- | --- | --- | --- | --- | --- | --- | --- | --- |
| MLP | 0.101 | 0.882 (0.811-0.938) | 0.902 (0.879-0.923) | 0.667 (0.456-0.861) | 0.911 (0.890-0.933) | 0.219 (0.125-0.324) | 0.987 (0.977-0.995) | 0.031 (0.020-0.043) | -0.007 | 0.963 |
| SVM | 0.064 | 0.872 (0.802-0.930) | 0.790 (0.757-0.821) | 0.792 (0.611-0.948) | 0.790 (0.756-0.822) | 0.123 (0.069-0.178) | 0.990 (0.982-0.998) | 0.032 (0.020-0.044) | -0.016 | 0.817 |
| RandomForest | 0.065 | 0.871 (0.794-0.934) | 0.806 (0.778-0.833) | 0.833 (0.667-0.964) | 0.805 (0.776-0.834) | 0.138 (0.082-0.196) | 0.992 (0.985-0.998) | 0.031 (0.020-0.042) | 0.021 | 0.840 |
| Logistic | 0.062 | 0.866 (0.786-0.928) | 0.800 (0.769-0.830) | 0.792 (0.603-0.939) | 0.801 (0.769-0.830) | 0.129 (0.072-0.185) | 0.990 (0.982-0.998) | 0.032 (0.020-0.043) | 0.053 | 0.778 |
| LightGBM | 0.034 | 0.865 (0.796-0.921) | 0.730 (0.694-0.762) | 0.917 (0.789-1.000) | 0.723 (0.684-0.758) | 0.110 (0.066-0.154) | 0.996 (0.989-1.000) | 0.031 (0.020-0.042) | -0.003 | 0.871 |
| CatBoost | 0.089 | 0.853 (0.771-0.919) | 0.797 (0.766-0.828) | 0.833 (0.667-0.960) | 0.796 (0.764-0.828) | 0.132 (0.076-0.189) | 0.992 (0.985-0.998) | 0.032 (0.021-0.043) | 0.002 | 0.902 |
| XGBoost | 0.124 | 0.848 (0.750-0.924) | 0.742 (0.710-0.775) | 0.875 (0.714-1.000) | 0.737 (0.703-0.772) | 0.111 (0.068-0.156) | 0.994 (0.987-1.000) | 0.042 (0.031-0.053) | 0.003 | 0.826 |
| GBDT | 0.045 | 0.847 (0.767-0.916) | 0.766 (0.736-0.799) | 0.833 (0.667-0.964) | 0.763 (0.732-0.797) | 0.116 (0.071-0.167) | 0.992 (0.984-0.998) | 0.031 (0.020-0.043) | -0.001 | 0.817 |
| AdaBoost | 0.459 | 0.832 (0.755-0.903) | 0.644 (0.608-0.681) | 0.917 (0.787-1.000) | 0.634 (0.594-0.671) | 0.086 (0.053-0.124) | 0.995 (0.988-1.000) | 0.196 (0.193-0.198) | 0.021 | 0.505 |
| KNN | 0.071 | 0.769 (0.661-0.864) | 0.808 (0.778-0.838) | 0.667 (0.460-0.860) | 0.813 (0.784-0.843) | 0.118 (0.058-0.177) | 0.985 (0.974-0.994) | 0.037 (0.025-0.050) | 0.081 | 0.941 |

Abbreviations: AUROC, area under the receiver operating characteristic curve; PPV, positive predictive value; NPV, negative predictive value; CI, confidence interval; AdaBoost, adaptive boosting; CatBoost, categorical boosting; XGBoost, extreme gradient boosting; RandomForest, random forest; LightGBM, light gradient boosting machine; GBDT, gradient boosting decision tree; KNN, k-nearest neighbors; MLP, multilayer perceptron; SVM, support vector machine.
