# Supplementary figures and images for: Association between blood cadmium and prevalent coronary heart disease in NHANES 2013 to 2014: A cross-sectional study with machine-learning analyses
Source: Medicine (Baltimore). 2026 Jul 3;105(27):e49554. doi: 10.1097/MD.0000000000049554 (PMC13337058; doi:10.1097/MD.0000000000049554)

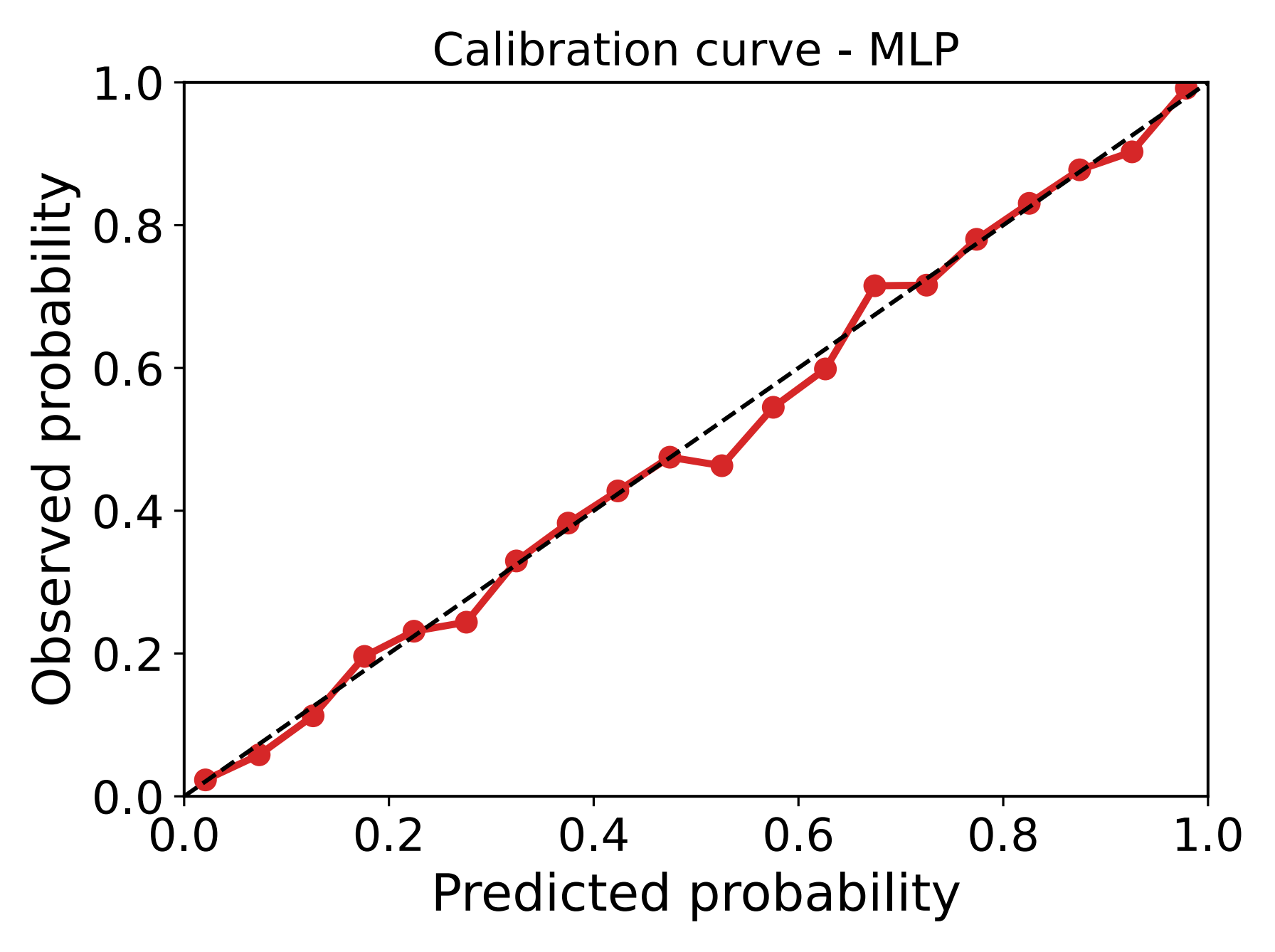

Supplement: Supplementary file 4 [file medi-105-e49554-s004.tif]
